# Probable Bruxism and Psychological Issues among Dental Students in Serbia during the COVID-19 Pandemic

**DOI:** 10.3390/ijerph19137729

**Published:** 2022-06-23

**Authors:** Veljko Kolak, Maja Pavlovic, Ema Aleksic, Vladimir Biocanin, Milica Gajic, Ana Nikitovic, Marija Lalovic, Irena Melih, Dragana Pesic

**Affiliations:** Faculty of Dentistry in Pancevo, University Business Academy in Novi Sad, 26000 Pancevo, Serbia; maja.pavlovic@sfp.rs (M.P.); ema.aleksic@sfp.rs (E.A.); vladimir.biocanin@sfp.rs (V.B.); milica.gajic@sfp.rs (M.G.); ana.nikitovic@sfp.rs (A.N.); marija.lalovic@sfp.rs (M.L.); irena.melih@sfp.rs (I.M.); dragana.pesic@sfp.rs (D.P.)

**Keywords:** COVID-19, bruxism, depression, anxiety, stress, fear, salivary cortisol, dental students

## Abstract

The COVID-19 pandemic has drastically changed the routine way of life, having consequences in many segments of life, including dental practice and education. The aim of this study was to evaluate the frequency of probable bruxism in a sample of dental students in Serbia and to estimate the potential association between psychological factors related to the COVID-19 pandemic and the presence of bruxism. A cross-sectional study included 178 dental students in Serbia, who were interviewed using a specially-designed self-administered online questionnaire, which consisted of three sections, and after that, a clinical examination for the presence of bruxism symptoms in the oral cavity. Psychological status was evaluated using the Depression, Anxiety and Stress Scale-21 (DASS-21) and the Fear of COVID-19 Scale (FCV-19S). Saliva samples were taken to analyze salivary cortisol levels. The prevalence of probable bruxism was 34.8%. Respondents with probable bruxism had significantly higher DASS-21 and FCV-19S scores and mean values of salivary cortisol compared to non-bruxers. A history of COVID-19 infection, high stress, and fear of COVID-19 scores were associated with the presence of probable bruxism. The findings suggest that the COVID-19 pandemic has had a great psychological impact and impact on the presence and worsening of bruxism symptoms in a sample of dental students in Serbia.

## 1. Introduction

Bruxism is a parafunctional habit described as a repetitive jaw-muscle activity characterized by clenching or grinding of the teeth and/or by bracing or thrusting of the mandible. It is called sleep bruxism (SB) when it occurs during sleep and awake bruxism (AB) when it occurs during wakefulness [1]. This condition was first described in the literature at the beginning of the 20th century and has been the subject of numerous studies since then [2]. Later on, Lobbezoo et al. made revised the definition and suggested that AB and SB should be considered different behaviors that occur during wakefulness and during sleep and that bruxism should not be considered as a disorder in otherwise healthy individuals but rather as a behavior or a physiological phenomenon, that could pose a risk factor for certain clinical consequences [3]. Studies revealed a number of factors associated with the occurrence of bruxism, such as teeth occlusion interferences, central or pathophysiological factors, and psychological factors, particularly stress. Previous studies also reported the association between bruxism and clinical signs, such as temporomandibular joint (TMJ) pain, TMJ noise, articular disc dislocation, osteoarthritis, limited mouth opening and abnormal opening pathway, presence of noticeable tooth wear, and increased tooth sensitivity [4,5,6]. Due to variations in demographics and the dependence on anamnestic data, the true prevalence of bruxism in any specific population is still unknown [7]. The reported prevalence of bruxism varied from 5% to 96%, and this wide variance was attributed to the definition used, diagnostic criteria applied, type of population sampled, types of questionnaires, and design of the study [8]. The literature reports an increased incidence of self-reported bruxism among university students from 5% to 22% over the period from 1966 to 2002 [9].

After the appearance of the first cases of pneumonia in Wuhan, China, and after the severe acute respiratory syndrome coronavirus 2 (SARS-CoV-2) was identified as the cause, due to a rapid increase in the number of cases outside China, in March 2020, the World Health Organization (WHO) announced that the outbreak could be characterized as a pandemic. Very soon, the European region became the epicenter of the respiratory coronavirus disease 2019 (COVID-19) epidemic, with over 40% of the globally confirmed cases [10]. All around the world, the pandemic has resulted in a number of problems, in the first place because of the numerous deaths and also because of the negative social and economic impacts. People’s daily routine was changed. Social distancing, school closure, changes in business, and travel bans significantly affected daily life. The pandemic also resulted in negative changes in health attitudes and physical activity, poorer sleep quality, and higher levels of depression, stress, and anxiety [11]. In Serbia, nine days after the first COVID-19 case was officially registered in March of 2020, a state of emergency was declared. The authorities implemented some of the European strictest measures, such as complete bans on movement, which sometimes lasted for several days. All cultural institutions, schools, and universities were suspended, and students were learning online, staying at home for days, weeks, or months [12]. The results of previous studies showed that students demonstrated a higher psychological impact from COVID-19 than those who were employed, possibly because of the prolonged school closure, online education, and uncertainty about exams. Students were also likely to experience fear of becoming ill or dying, feelings of helplessness, and stigma [13]. COVID-19 pandemic also massively affected clinical dental education and clinical dental practice as well [14].

Although bruxism is a common issue with a relatively high prevalence reported in previous studies, there is still a lack of epidemiological data about the influence of the COVID-19 pandemic on that condition, especially in a vulnerable population such as university students. The aim of this study was to evaluate the frequency of probable bruxism in the sample of dental students in Serbia and to estimate the potential association between psychological factors related to the COVID-19 pandemic and the presence of bruxism.

## 2. Materials and Methods

A cross-sectional study was conducted on a sample of dental students from the Faculty of Dentistry in Pancevo, Serbia, approximately a year and a half since the beginning of the COVID-19 pandemic. A questionnaire through an electronic form developed on the Google Forms platform was sent during the semester break to 300 active dental students who, according to the faculty records, attended classes on a regular basis enrolled on a five-year study program. The self-administered questionnaire consisted of three sections: (1) sociodemographic characteristics and general health (age, gender, year of study, phone number, presence of any chronic disease, and current medication), (2) section about COVID -19 experience (history of infection with COVID-19, were they hospitalized because of the present symptoms, were they in isolation because of the close contact with the infected person, was some close family member infected and hospitalized, are they vaccinated), (3) section about the presence of bruxism (if they are aware of the fact grinding or clenching their teeth during sleep or when awake, have they ever been told that they grind their teeth during sleep, if they have their jaws thrust or braced after they wake up in the morning or during the night and if they have noticed worsening of symptoms since the beginning of the pandemic).

A test–retest correlation on a preliminary sample of subjects at two distinct time periods was used to test the reliability of the questionnaire. The correlation coefficient (r) was 0.89, which was considered good.

**Figure 1:**
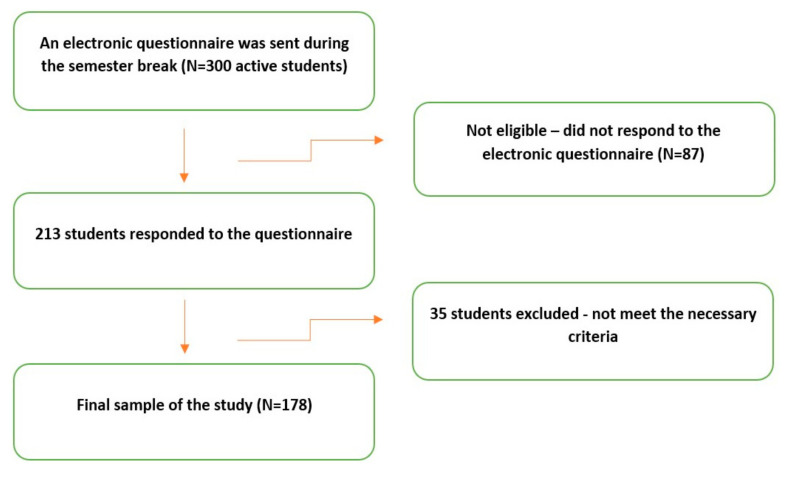
A flowchart of the recruitment process.

A preliminary electronic questionnaire (Supplementary File S1) was completed by 213 students. After the beginning of the new semester, the participants were appointed for further examination. The moment was chosen since it was far from any seasonal holidays and stressful periods due to exams. The inclusion criteria for participating in the study were willing to participate in the study and the absence of systematic diseases. The exclusion criteria were an unwillingness to participate in the study, presence of some severe chronic illness, cognitive disability and active inflammation, history or presence of severe neuromuscular illness, ongoing medication with antipsychotic, antidepressant drugs, hormonal therapy, and contraceptive pills, more than two missing teeth per quadrant (excluding third molars), ongoing prosthodontic or orthodontic treatment. After excluding those who did not meet the necessary criteria, the final number of respondents was 178 (Figure 1). The Raosoft, Inc. survey software (Seattle, WA, USA; http://www.raosoft.com, accessed on 21 July 2021) was used for the sample size calculation. Power analysis revealed that the study required at least 169 participants, with the margin of error set at 5% with 95% confidence intervals. The study was conducted in complete accordance with the World Medical Association’s Declaration of Helsinki. The subjects were fully informed about the study and gave written consent to participate voluntarily. The investigation was approved by the Ethics Commission of the Faculty of Dentistry in Pancevo (Approval Protocol No. 689/2-2021, according to Resolution sections 3, 7, and 8 of the National Commission of Ethics in Research).

Each participant underwent an intraoral, and extraoral examination focused on finding bruxism symptoms in the oral cavity: indentation on the tongue and/or linea alba on the inner cheek, presence of noticeable tooth wear spots on the incisal surfaces of the anterior teeth and/or on the guiding cusps of the posterior teeth, increased tooth sensitivity, muscle palpation test, sounds associated with bruxism, jaw muscle discomfort, limited mouth opening. The presence of possible bruxism was based on self-report as recommended by Lobbezoo et al. [3] and later validated by clinical examination and presence of at least one clinical feature, as recommended by the American Academy of Sleep Medicine [15]. The clinical examination was performed by two previously trained and calibrated dentists following recommendations from the WHO for reliability and validity of data. After agreement concerning the diagnostic procedure and criteria was reached, a preliminary sample of 20 participants was examined separately by each examiner two times. After examination, examiners compared their records and conclusions. In case of a disagreement, a mutual consensus was reached after a joint review. The inter-examiner agreement was calculated using Kappa statistics resulting from the preliminary examination. Cohen’s Kappa value index was 0.88, which is considered excellent.

The evaluation of the participant’s psychological status was undertaken using the Depression, Anxiety, and Stress Scale-21 (DASS-21), translated into the Serbian language. The DASS-21 is a shortened version of the original 42-item DASS created by Lovibond and Lovibond [16]. It is a self-report scale designed to estimate the overall emotional distress of a respondent which consists of 21 statements divided into three subscales (depression, anxiety, and stress), each containing 7 statements. The participants were instructed to write how well they agreed with each statement since the beginning of the COVID-19 pandemic. The answers for each statement were categorized on a 4-point Likert scale ranging from 0 to 3 (0—does not apply to me at all, 1—somewhat applies to me, 2—applies to me quite often and 3—applies to me most of the time or always). The sum of the 7 scores of each subscale multiplied by 2 determined its final score. The score for each subscale ranges between 0 and 42. Higher scores indicated greater levels of distress. As recommended by the original authors of the DASS-21 scale [16], the categorization for three subscales was as follows: depression (0–9 = normal, 10–13 = mild, 14–20 = moderate, 21–27 = severe and score 28+ = extremely severe), anxiety (0–7 = normal, 8–9 = mild, 10–14 = moderate, 15–19 = severe and score 20+ = extremely severe), stress (0–14 = normal, 15–18 = mild, 19–25 = moderate, 26–33 = severe and score 34+ = extremely severe). This scale has proven to be an accurate measure for levels of depression, anxiety, and stress during the COVID-19 outbreak in various countries [13,17,18] and was also previously validated on the Serbian student population [19], as well as on the general adult population of Serbia since the beginning of COVID-19 pandemic [12,20].

Every participant of the study also completed the Fear of COVID-19 Scale (FCV-19S), developed, and initially validated by Ahorsu et al. [21] and later validated in countries across the world [22,23,24]. The questionnaire was translated and back-translated to ensure that the expressions were appropriate, according to the recommendations by the WHO, such as in previous studies conducted in Serbia [25,26]. FCV-19S is a seven-item self-administered uni-dimensional scale with answers rated on a five-point scale from 1 (completely disagree) to 5 (strongly agree). A total score is calculated by adding up each item’s score (ranging from 7 to 35). The higher the score, the greater the fear of COVID-19. For the purpose of this study, the overall scores were categorized into low and high levels of fear, taking the mean values as the cut-off, similar to Giordani et al. study [27].

Saliva samples were taken from all subjects with probable bruxism (self-reported and later validated by clinical examination) and the same number of subjects without any signs of bruxism and temporomandibular dysfunction (control group) to analyze salivary cortisol levels as a potential indicator of stress. Saliva samples were collected at room temperature in the middle of the class day (2 pm) on Wednesday (middle of the class week). A day before the collection of saliva, the participants were instructed not to have any alcohol less than 12 h before, not to take food or fluids for 60 min before the collection, and not to take anything with caffeine (coffee, tea, soda, chocolate) for 2 h before the collection, not to apply creams or lotions that contain steroids right before the collection and not to brush or floss teeth or do activities that may cause the gums to bleed. As recommended by the lab, 2 mL of unstimulated saliva were collected from the participants by asking them to spit in a salivette, also provided by the lab. A similar procedure was undertaken in previous studies [28,29]. The participants’ names, date of birth, and the time of sample collection were written on the label, and all of the samples were transported to the lab for analysis immediately after collection, so there was no need to keep them refrigerated. The cortisol levels in saliva were measured in the lab using the ELISA immunoassay test, Eclia, which is based on the competitive binding of specific cortisol polyclonal antibodies. The cortisol concentration was expressed in nmol/L.

The collected data were analyzed using statistical software SPSS v20.0 (IBcM Inc., Armonk, NY, USA). The descriptive statistics for the distribution of respondents based on questionary answers were expressed as numbers and percentages for all categories. The results of DASS-21 and FCV-19S scores were expressed as percentages, with mean values, standard deviation, and minimum and maximum values. An independent *t*-test was used to evaluate the difference in DASS-21 and FCV-19S scores between probable bruxers and non-bruxers, at a significance level of *p* ≤ 0.05. Wilcoxon signed-rank test was used to analyze the difference in salivary cortisol concentrations between probable bruxers and non-bruxers (significance level *p* ≤ 0.05). Subject-level analysis was conducted to evaluate the association between possible variables and the presence of probable bruxism using logistic regression. Each variable was first employed as an independent variable in a univariate unconditional logistic regression, with the presence of probable bruxism as a dependent variable. Variables with significant correlation were then used as independent variables in the multivariate logistic analysis. The strength of association was presented by an odds ratio (OR) at a significance level of *p* ≤ 0.05 with a 95% Confidence interval (CI).

## 3. Results

### 3.1. Distribution of Respondents Based on Questionary Answers

The research included 178 university students, 94 females and 84 males. The youngest respondent was 19, the oldest one was 30, and the average age of the study sample was 23.1. At the time of completing the questionnaire, 39.3% of respondents declared that they were in some period of time infected with the SARS-CoV-2 (COVID-19) virus and the vast majority (84.3%) of them had some close family member who was infected. The presence of sleep or awake bruxism was reported by 34.8% of respondents, and 74.2% of them declared that they had noticed symptoms worsening since the beginning of the COVID-19 pandemic. The distribution of respondents based on questionary answers is presented in Table 1.

### 3.2. Psychological Status Evaluation

The results of the evaluation of the psychological indicators during the COVID-19 pandemic in the study sample using DASS-21 and Fear of COVID-19 (FCV-19S) scales are displayed in Table 2.

The results for the depression, anxiety, and stress subscales of DASS-21 showed that the largest number of respondents (71.9%, 70.8%, and 69.7%, respectively) had normal scores, while 4.5%, 10.1%, and 6.6%, respectively, had severe or extremely severe scores (Table 3).

### 3.3. Bruxism and Psychological Factors

Independent *t*-test analysis showed that respondents with probable sleep or awake bruxism had statistically significant higher scores for depression, anxiety, and stress subscales of DASS-21 and FCV-19S compared to the respondents without the presence of probable bruxism (Table 4).

Table 5 shows the mean values of salivary cortisol concentrations, as a possible stress indicator, among respondents with probable sleep or awake bruxism and the control group (non-bruxers). Wilcoxon signed-rank test showed a significant difference between the two groups of respondents (*p* < 0.001).

The association of factors related to the COVID-19 pandemic from the questionnaire, as well as gender and DASS-21 and FCV-19S scores with the presence of probable bruxism, was analyzed using logistic regression. The results of the univariate unconditional logistic regression showed a direct link between the presence of probable bruxism and gender (*p* < 0.001), history of COVID-19 infection (*p* < 0.001), history of close family members infection (*p* = 0.019), high depression (*p* = 0.004), anxiety (*p* = 0.005), and stress (*p* < 0.001) score, as well as with high fear of COVID-19 score (*p* < 0.001). After conducting the multivariate logistic regression analysis, history of COVID-19 infection (*p* < 0.001), high-stress score (*p* = 0.004), and high fear of COVID-19 score (*p* = 0.001) were associated with the presence of probable sleep or awake bruxism (Table 6).

## 4. Discussion

After a state of emergency was declared because of the COVID-19 pandemic, all faculties in Serbia, among them the Faculty of Dentistry in Pancevo, have indefinitely suspended physical lecture attendance, clinical and preclinical practical activities, and exams, respecting recommendations by the authorities for monitoring the epidemiological situation and coordinating further measures in accordance with the general situation in the country. Moreover, among other restrictions, dental offices in each city were instructed to limit their practice only to emergencies in daily time when a complete ban on movement was not declared. After the beginning of the new academic semester, the majority of Serbian schools of dentistry started using a hybrid system, with lectures being held online, while practical clinical activity was held physically on-site, in a small group of students, and with a minimum number of patients, with strict implementation of all epidemiological measures. Since the beginning of the COVID-19 pandemic, a number of studies have evaluated the impact of the pandemic on students’ emotional health status, fear, anxiety disorders, depression, and stress [13,17,30,31,32]. The pandemic impact on dental education is stated to be more destructive, mainly because of the fact that the educational process involves the gain of practical skills, which is performed by the students directly on the patient [33].

Until June of 2021, a total of 714,753 COVID-19 cases had been registered in Serbia, including 159,651 cases in the Autonomous Province of Vojvodina, where the city of Pancevo belongs [34]. At the moment of preliminary questioning (which was a year and a half since the beginning of the pandemic), 39.3% of students reported that they had been infected with COVID-19 (tested positive), and 3 of 70 cases had been hospitalized because of severe clinical symptoms. The vast majority of students reported that they had a family member infected (84.3%) and experienced isolation because of the close contact with the infected person (84.8%). Those results are not easy to compare with the results of other studies, namely due to the lack of accurate data and the difference between the time period in which they were conducted. In a study among 4355 college students in Wuhan, China, conducted 3 months after the beginning of the pandemic, the reported prevalence of infected students was 1.6% [35]. In a large study that examined self-reported COVID-19 infection rates among 100,488 American college students enrolled during the fall of 2020, the reported prevalence was 6.79% [36], and a similar percentage (6%) for the same period was reported among students in Turkey [37].

The literature broadly describes the prevalence of bruxism. However, the indirect comparisons from the literature should be interpreted with caution because diagnosis methods, clinical criteria, and samples of the population varied between studies [4]. Manfredini et al., in a systematic review of the literature, reported the prevalence of bruxism ranking from 8 to 31.4% in adults [38]. It should be noted that a higher bruxism prevalence among students with respect to the data on the general population appeared to be consistent with current literature [39]. The presence of probable sleep or awake bruxism was recorded among 34.8% of the subjects in the present study. This result is consistent with the results of recent studies also conducted among dental students, with the prevalence of bruxism ranking from 23.7 to 49.2% [40,41,42].

The long-lasting pandemic situation and strict measures negatively affected higher education and the mental health of university students, who exhibited increased levels of fear, stress, anxiety, and depressive thoughts [31]. The mean values for depression, anxiety, and stress recorded in this study were lower than in studies that examined university students in the USA, Poland, and Bangladesh. Moreover, the percentage of respondents who exhibited at least moderate levels according to DASS-21 categories (12.4%, 25.8%, and 16.9%, respectively) was lower than in a study conducted among Bangladeshi university students [30,43,44]. It must be emphasized that all of these studies were conducted early in the COVID-19 outbreak so that the results could be expected due to partial adaptation to the pandemic after a year and a half. Nevertheless, the mean values of DASS-21 scores recorded in the present study were higher than in studies conducted in Spain and Ghana, two months after the beginning of the pandemic [43,45]. The recorded level of fear of COVID-19 measured using the FCV-19S scale in the present study was also lower than in previous studies among university students [45,46,47]. In addition to the stated reason regarding the different time periods when studies were conducted, the reasons for such results can be found in the different number of infected people, deaths, and measures of total lockdown in different countries. It is likely that the higher number of infections and deaths in the first months of the first wave of the pandemic and the strict lockdown orders could have generated higher levels of fear of COVID-19 [26].

The results of the present study showed that respondents with probable bruxism had statistically significant higher scores for the depression, anxiety, and stress subscales of DASS-21 and FCV-19S compared to the respondents without the presence of bruxism. In addition, 74.2% of bruxers declared that they had noticed symptoms worsening since the beginning of the COVID-19 pandemic. Such a result was also obtained in the Yıldırım et al. study performed among Turkish dental students [48]. Previous studies demonstrated the relationship between bruxism and psychosocial factors. The pandemic further contributes due to the nature of quarantine and subsequent isolation, which is also an established risk factor for psychological impact. The new dimension is certainly a concern for family members and close friends and their potential infection. Due to the altered living conditions during the pandemic, many of the identified risk factors tend to increase [49]. Depression, anxiety, and stress associated with a stressful event, such as the current pandemic, can exacerbate bruxism. Participants without symptoms of depression, anxiety, and stress were less likely to report bruxism [50].

Cortisol, the stress hormone, is considered a potential marker of psychological status and thus could be correlated with an anxious and stressed status and the presence of bruxism. The results of the present study showed significantly higher levels of salivary cortisol among bruxers compared to non-bruxers. Similar results were reported in Fluerașu et al. and Khayamzadeh et al. studies [51,52]. The major advantage of such analysis is that samples can be obtained outside the laboratory and the fact that the collection of saliva is a non-invasive sampling method that does not induce additional stress in participants [53].

Multiple logistic regression models revealed that the presence of probable bruxism was associated with a history of COVID-19 infection, a high-stress score, and a high fear of COVID-19 score. A review article, performed about 10 months after the onset of the pandemic, found only a few studies dealing with temporomandibular disorders (TMD) and bruxism during COVID-19. The majority of them revealed adverse effects on participants’ psycho-emotional status (stress, anxiety, and depression), which in turn led to the intensification of TMD and bruxism symptoms and increased orofacial pain [10]. Oh et al., in their study, reported that COVID-19 infection might be associated with a risk of psychosis [54]. COVID-19 is characterized by high infectivity and low mortality, but those who contracted the virus at the onset of the pandemic faced uncertain prognosis after a long time of suffering, the fear of infecting others, expenses for medical care, and probable stigmatization and exclusion. All those factors may act as triggers of psychosis symptoms among infected persons [55]. As mentioned before, elevated levels of stress have a well-established link to bruxism [39]. Studies prior to the pandemic reported that university students suffer from higher levels of depression and anxiety than the general population. Moreover, levels of depression and anxiety have been higher among dental students than among university students in general, particularly since the beginning of the pandemic [56,57]. In addition to the higher scores for depression and anxiety among bruxers in the present study, the multiple logistic regression model did not reveal a significant association. Similar results were presented in Goulart et al. study [58]. Moreover, in addition to the higher percentage of bruxers among female respondents in the present study, gender was not significantly associated with the occurrence of bruxism. Some authors reported that the female gender played a significant role in predicting the presence of bruxism. The offered explanation was that women are more vulnerable to the effects of unexpected, prolonged stress situations than men [59]. The results of two recent studies are suggesting that the prevalence does not vary between genders [60] or that the male gender was indicated as a risk factor [61].

Some limitations of the study should be highlighted. The study considered probable bruxism, which is based on a positive clinical inspection with a positive self-report. It should be mentioned that for definite bruxism, a positive instrumental assessment should be provided. Electromyographic (EMG) recordings during wakefulness may provide key evidence for the presence of bruxism. Furthermore, SB and AB were not assessed separately, which could have led to even more accurate data. All of the subjects included in the study were students from the same university, so the study sample could not be considered nationally representative. Since a cross-sectional study addresses the present time only, there is a possibility that the situation has changed since the beginning of the pandemic. It is a fairly new topic; limited research has been conducted, and further longitudinal studies should be conducted to understand the relationship between COVID-19 stressors and orofacial disorders.

## 5. Conclusions

Within the limitations of this study, the prevalence of probable bruxism (AB and SB together) among dental students in Serbia was 34.8%. A history of COVID-19 infection, high stress, and the fear of COVID-19 scores were associated with the presence of probable bruxism. Since COVID-19-related stressors have led to the worsening of bruxism symptoms, giving advice on how to cope with stress and providing interventions such as mouth guards could help minimize the risk of negative consequences.

## Figures and Tables

**Table 1:** Distribution of respondents based on questionary answers.

Variable	Category	Total Number (N)	Percentage (%)
Gender	Female	94	52.8
	Male	84	47.2
Been infected with COVID-19	Yes	70	39.3
	No	108	60.7
Been hospitalized	Yes	3	1.7
	No	175	98.3
Been isolated	Yes	151	84.8
	No	27	15.2
A family member has been infected	Yes	150	84.3
	No	28	15.7
A family member has been hospitalized	Yes	28	15.7
	No	150	84.3
Fully vaccinated	Yes	56	31.5
	No	122	68.5
Self-reported sleep or awake bruxism	Yes	62	34.8
	No	116	65.2
Worsening of bruxism symptoms	Yes	46	74.2
	No	16	25.8

**Table 2:** DASS-21 and FCV-19S scores of the study sample.

Scale	Category	Mean ± SD	Range
DASS-21	Depression	6.04 ± 6.18	0–28
	Anxiety	5.66 ± 7.24	0–36
	Stress	10.87 ± 8.27	0–36
	Total	22.57 ± 19.09	0–94
FCV-19S			
	Total	12.56 ± 4.49	7–28

**Table 3:** Distribution of respondents according to DASS-21 categories.

Variable	Category	Total Number (N)	Percentage (%)
Depression	Normal	128	71.9
	Mild	28	15.7
	Moderate	14	7.9
	Severe	6	3.4
	Extremely severe	2	1.1
Anxiety	Normal	126	70.8
	Mild	6	3.4
	Moderate	28	15.7
	Severe	8	4.5
	Extremely severe	10	5.6
Stress	Normal	124	69.7
	Mild	24	13.5
	Moderate	19	10.7
	Severe	9	5.1
	Extremely severe	2	1.1

**Table 4:** Difference of DASS-21 and FCV-19S scores between bruxers and non-bruxers.

Variable	Bruxers		Non Bruxers			
	Mean	SD	Mean	SD	*t* Value	*p*
DASS-21 Depression	8.06	7.94	4.97	4.68	3.2759	0.001 *
DASS-21 Anxiety	9.29	9.83	3.72	4.31	5.2374	<0.001 *
DASS-21 Stress	14.87	8.74	8.72	7.17	5.0403	<0.001 *
DASS-21 Total	32.23	23.63	17.41	13.71	5.2945	<0.001 *
FCV-19S	15.06	4.92	11.22	3.61	5.9366	<0.001 *

* Significant at *p* < 0.05.

**Table 5:** Difference of salivary cortisol concentrations between bruxers and non-bruxers.

Group	Salivary Cortisol (nmol/L)			
	Mean	SD	z Value	*p*
Bruxers	10.99	7.25	−4.6098	<0.001 *
Non-bruxers	6.65	1.84		

* Significant at *p* < 0.05.

**Table 6:** Analysis of variables associated with probable bruxism.

Variable	Presence of Bruxism		Univariate Logistic Regression Analysis			Multivariate Logistic Analysis		
	N	%	OR	95% CI	*p*-Value	OR	95% CI	*p*-Value
Gender								
Female	46	48.9	4.073			1.760		
Male	16	19.0	1	2.07–8.03	˂0.001 *	1	0.71–4.36	0.223
Been positive for COVID-19								
No	18	16.7	0.118			0.092		
Yes	44	62.9	1	0.06–0.24	˂0.001 *	1	0.04–0.23	˂0.001 *
Hospitalized								
No	60	34.3	0.261					
Yes	2	66.7	1	0.02–2.94	0.277			
Isolated								
No	5	18.5	0.375					
Yes	57	37.7	1	0.13–1.04	0.061			
Family member has been infected with COVID-19								
No	4	14.3	0.264			0.382		
Yes	58	38.7	1	0.09–0.80	0.019 *	1	0.09–1.53	0.174
Family member hospitalized								
No	50	33.3	0.667					
Yes	12	42.9	1	0.29–1.52	0.334			
DASS21 Depression								
Normal/Mild	48	30.8	0.254			0.618		
Mod.-Severe	14	63.6	1	0.10–0.65	0.004 *	1	0.11–3.38	0.579
DASS21 Anxiety								
Normal/Mild	38	28.8	0.371			0.723		
Mod.-Severe	24	52.2	1	0.19–0.74	0.005 *	1	0.23–2.23	0.572
DASS21 Stress								
Normal/Mild	42	28.4	0.198			0.136		
Mod.-Severe	20	66.7	1	0.09–0.46	˂0.001 *	1	0.03–0.53	0.004 *
FCV-19S								
Low	18	18.7	0.199			0.242		
High	44	53.7	1	0.10–0.39	˂0.001 *	1	0.11–0.55	0.001 *

* Significant at *p* < 0.05.

## Data Availability

The data presented in this study are available upon request from the corresponding author.

## Supplementary Materials

The following supporting information can be downloaded at: https://www.mdpi.com/article/10.3390/ijerph19137729/s1. Supplementary File S1: A preliminary electronic questionnaire.
