# Supplementary material for: Probable Bruxism and Psychological Issues among Dental Students in Serbia during the COVID-19 Pandemic
Source: Int J Environ Res Public Health. 2022 Jun 23;19(13):7729. doi: 10.3390/ijerph19137729 (PMC9266173; doi:10.3390/ijerph19137729)
Supplement: Supplementary file 1 [file ijerph-19-07729-s001.zip › ijerph-1764326-supplementary.pdf]

Questionnaire

**SECTION A**		
**1.**	**Name**	
**2.**	**Gender**	
**3.**	**Age**	
**4.**	**Year of study**	
**5.**	**Phone number**	
**6.**	**Presence of any chronic disease?**	
**7.**	**Are you currently taking some medication?**	
**SECTION B**		
**1.**	**Have you been tested positive for the presence of Covid 19?**	
**2.**	**If yes, have you been hospitalized because of the present symptoms?**	
**3.**	**Have you been in isolation because of the close contact with the infected person?**	
**4.**	**Was some close family member of yours infected with Covid 19?**	
**5.**	**Was some close family member of yours hospitalized because of the present symptoms?**	
**6.**	**Have you been vaccinated against Covid 19 virus infection?**	
**SECTION C**		
**1.**	**Are you aware of the fact of grinding or clenching your teeth during sleep or when awake?**	
**2.**	**Have you ever been told that you grind your teeth during sleep?**	
**3.**	**Do you have your jaws thrust or braced after you wake up in the morning or during the night?**	
**4.**	**Have you noticed a worsening of symptoms since the beginning of the Covid 19 pandemic?**	
